# RETRACTION: Effect of drain placement in short‐level spinal surgery on postoperative wound infection: A meta‐analysis

**DOI:** 10.1111/iwj.70112

**Published:** 2024-10-16

## Abstract

Retraction: ZhanB. , FangS. , LvX. , XieX. , WangX. , “Effect of Drain Placement in Short‐Level Spinal Surgery on Postoperative Wound Infection: A Meta‐Analysis,” International Wound Journal 21, no. 3 (2024): e14508, 10.1111/iwj.14508.38037852 PMC10898379

The above article, published online on 01 December 2023, in Wiley Online Library (http://onlinelibrary.wiley.com/), has been retracted by agreement between the journal Editor in Chief, Professor Keith Harding; and John Wiley & Sons, Ltd. It came to the publisher's attention from a third party that a number of articles shared concerning similarities in format and structure. Following an investigation by the publisher, the retraction has been agreed on as the peer review and publishing process for this article were found to be manipulated. The authors did not respond to the notice of retraction.

Retraction: B. Zhan , S. Fang , X. Lv , X. Xie , X. Wang , “Effect of Drain Placement in Short‐Level Spinal Surgery on Postoperative Wound Infection: A Meta‐Analysis,” International Wound Journal 21, no. 3 (2024): e14508, 10.1111/iwj.14508.38037852 PMC10898379

The above article, published online on 01 December 2023, in Wiley Online Library (http://onlinelibrary.wiley.com/), has been retracted by agreement between the journal Editor in Chief, Professor Keith Harding; and John Wiley & Sons, Ltd. It came to the publisher's attention from a third party that a number of articles shared concerning similarities in format and structure. Following an investigation by the publisher, the retraction has been agreed on as the peer review and publishing process for this article were found to be manipulated. The authors did not respond to the notice of retraction.

